# *MBL-2* polymorphisms (codon 54 and Y-221X) and low MBL levels are associated with susceptibility to multi organ dysfunction in *P. falciparum* malaria in Odisha, India

**DOI:** 10.3389/fmicb.2015.00778

**Published:** 2015-07-31

**Authors:** Bidyut K. Das, Aditya K. Panda

**Affiliations:** ^1^Department of Medicine, SCB Medical CollegeCuttack, India; ^2^Centre for Life Sciences, Central University of JharkhandRanchi, India

**Keywords:** *MBL2*, gene polymorphism, *P. falciparum*, plasma MBL, association

## Abstract

**Background:** Mannose binding lectin, a plasma protein protects host from virus, bacteria, and parasites. Deficiency in MBL levels has been associated with susceptibility to various infectious diseases including *P. falciparum* malaria. Common MBL polymorphisms in promoter and coding regions are associated with decrease in plasma MBL levels or production of deformed MBL, respectively. In the present study, we hypothesized that *MBL2* variants and plasma MBL levels could be associated with different clinical phenotypes of severe *P. falciparum* malaria.

**Methods:** A hospital based study was conducted in eastern Odisha, India which is endemic to *P. falciparum* malaria. Common *MBL-2* polymorphisms (codon 54, H-550L, and Y-221X) were typed in 336 cases of severe malaria (SM) [94 cerebral malaria (CM), 120 multi-organ dysfunction (MOD), 122 non-cerebral severe malaria (NCSM)] and 131 un-complicated malaria patients (UM). Plasma MBL levels were quantified by ELISA.

**Results:** Severe malaria patients displayed lower plasma levels of MBL compared to uncomplicated falciparum malaria. Furthermore, on categorization of severe malaria patients into various subtypes, plasma MBL levels were very low in MOD patients compared to other categories. Higher frequency of AB genotype and allele B was observed in MOD compared to UM (AB genotype: *P* = 0.006; B allele: *P* = 0.008). In addition, prevalence of YX genotype of MBL Y-221X polymorphism was also statistically more frequent in MOD case than UM (*P* = 0.009).

**Conclusions:** The observations of the present study reveal that *MBL-2* polymorphisms (codon 54 and Y-221X) and lower plasma MBL levels are associated with increased susceptibility to multi organ dysfunctions in *P. falciparum* malaria.

## Introduction

Malaria is a mosquito borne protozoan infection caused by *Plasmodium falciparum, P. vivax, P. ovale, P. malariae*, and *P. knowlesi*. In a recent report, World Health Organization (WHO) estimates about198 million cases in 2013 and 1.2 billion subjects are at high riskworldwide (WHO, 2014). Majority of malarial death is a result of *P. falciparum* infection which leads to severe malaria characterized by cerebral malaria (CM), multi-organ dysfunction (MOD), and non-cerebral severe malaria (NCSM) (Buffet et al., 2011; Panda et al., 2011, 2012; Pattanaik et al., 2012). The pathogenesis of malaria is complex and the severity depends on parasite virulence, transmission dynamics, genetic factors, and host immune response (Buffet et al., 2011; Mangano and Modiano, 2014; White et al., 2014). Malaria infection elicits both innate and adaptive immune response of the host. The innate immune system is composed of diverse pattern-recognizing receptors or soluble pathogen-recognizing molecules (PRMs) (Thiel et al., 2006; Barreiro et al., 2009), which recognize specific molecular motifs on the surfaces of virus, bacteria and parasites. Mannose binding lectin is a liver derived soluble PRMs which play an important role in the innate immune response. MBL binds to sugars on the surface of pathogenic micro-organisms and triggers the complement activation system (Ip et al., 2009). MBL has been shown to bind to parasite infected erythrocytes (Garred et al., 2003b) and children deficient in MBL are prone to severe malaria (Luty et al., 1998), indicating an important role for MBL in protection against *P. falciparum* malaria.

The human MBL is a 32 KDa protein consisting of 248 amino acids encoded by *MBL2* mapped to 10q21.1 (Garred et al., 2006). Common MBL2 genetic variants have been associated with various diseases such as filariasis (Choi et al., 2001; Meyrowitsch et al., 2010), malaria (Boldt et al., 2006; Jha et al., 2014), leishmaniasis (Asgharzadeh et al., 2007), leprosy (de Messias-Reason et al., 2007; Sapkota et al., 2010), tuberculosis (Singla et al., 2012; Chen et al., 2014), trypanosomiasis (Weitzel et al., 2012), HIV infection(Li et al., 2013), systemic lupus erythematosus (Panda et al., 2013), and rheumatoid arthritis (Martiny et al., 2012). The MBL2 gene consists of four exons. Although several single nucleotide polymorphisms (SNPs) have been reported, three SNPs in exon 1 (codons 52: rs5030737, C > T, Arg>Cys; codon 54: rs1800450, G > A, Gly> Asp and codon 57: rs1800451, G >A,Gly>Glu) are of importance since they affect plasma levels of MBL. Variants are denoted as D (codon 52), B (codon 54), and C (codon 57), whereas A is the common wild type allele (Garred et al., 2006). In addition, two other functional polymorphisms at promoter region of the MBL2 gene have been reported (−550: rs11003125,G > C, H/L and−221: rs7096206, C > G, X/Y) which have been shown to affect plasma MBL levels (Garred et al., 2003a). Furthermore, some reports have shown an association of combined exon1 and promoter *MBL2* polymorphisms with plasma levels of MBL (Tsutsumi et al., 2001; Panda et al., 2013).

The association between *MBL2* polymorphism and *P. falciparum* malaria in Gabonese children has been reported in several studies but it has shown contradictory results. Luty et al. (1998), showed an association between codon 54 and 57 variants with susceptibility to severe malaria. On the contrary, another study failed to show similar association (Mombo et al., 2003). Interestingly, a novel mutation (−797*C* > A) has been linked to susceptibility to severe malaria (Boldt et al., 2006).

A recent study in Indian population revealed association between *MBL2* LYPA haplotype with protection and *MBL2* LXPA haplotype with increased susceptibility to severe malaria (Jha et al., 2014). To the best of our knowledge, there are no studies on the association of plasma MBL and common *MBL2* polymorphism with *P. falciparum* malaria in well-defined clinical phenotypes. We report an association between low plasma MBL and MBL low producer genotypes with multi organ dysfunction in Odisha, India.

## Materials and methods

### Study site and sample collection

In the present study, patients attending and/or admitted to Department of Medicine, SCB Medical College and Hospital, Cuttack, Odisha were included. Odisha is endemic for malaria and more than 85% cases are attributed to *P. falciparum* infection (Panda et al., 2011, 2012). Laboratory diagnosis of malarial infection was conducted by immunochromatography test (ICT) (SD Bio Standard Diagnostics India) and nested PCR assay (Panda et al., 2011). Patients who were positive for both the tests were included in the present study. PCR was done to exclude co-infection with *P. vivax*. There were no discrepancies for diagnosis of *P. falciparum* infection. Based on WHO guidelines and as described earlier (Panda et al., 2011, 2012; Pattanaik et al., 2012). Patients were categorized into two broad groups: (1) uncomplicated malaria (UM) and (2) severe malaria (SM). SM cases were further categorized into three sub-groups (i) Cerebral malaria (CM) defined as patients with altered sensorium, GCS (Glasgow Coma Scale) of ≤10; (ii) Non cerebral severe malaria (NCSM) patients had one of the several manifestations of severe malaria without cerebral involvement, namely severe anemia (hemoglobin < 5 g/dl), acute renal failure (serum creatinine>3 mg/dl), jaundice (serum bilirubin >3 mg/dl), acute respiratory distress syndrome (PaO2/ FIO2 < 200), haemoglobinuria (urine positive for hemoglobin), and shock (systolic BP of < 80 mm Hg);and (iii) Multi-organ-dysfuction (MOD) diagnosed based on presence of two or more organ involvement like CNS (GCS ≤10), respiratory (PaO_2_/FIO_2_ < 200), renal failure (serum creatinine>3 mg/dl), and hepatic dysfunction (ALT/AST > three times of normal, prolonged prothrombin time and low albumin). Patients with following criteria were excluded from current investigations: (i) Co-infection with other Plasmodium species, (ii) chronic disease like tuberculosis, chronic renal failure, cirrhosis of liver and autoimmune diseases like systemic lupus erythematosus and rheumatoid arthritis. Patients enrolled in the present study came from the coastal districts of the state having an average annual parasite index (API) of 6.67. 100 healthy controls were included from the same districts and selectively included so that both the groups are uniformly exposed during transmission of malaria. None of the controls reported history of clinical malaria in the last 5 years. They were essentially healthy and negative for demonstrable *P. falciparum* infection. Demographic characteristics of enrolled subjects are shown in Table 1. About 5 ml of venous blood was collected in EDTA vials from all enrolled patients and HC. The study and its protocols were approved by the Institutional Human Ethics Committee of SCB Medical College Cuttack. Blood samples were collected after obtaining written consent of the healthy controls and patients or accompanying person (in case of comatose patients).

**Table 1:** **Demographic characteristics of malaria patients enrolled in the study**.

**Characteristics**	**Clinical categories**				
	**CM (*n* = 94)**	**MOD (*n* = 120)**	**NCSM (*n* = 122)**	**UM (*n* = 131)**	**HC (*n* = 100)**
Male/Female	75/19	94/26	100/22	97/34	82/18
Age (mean ± S.D)	33.18 ± 14.74	34.63 ± 14.26	34.93 ± 13.68	33.66 ± 14.31	30.82 ± 14.50

*CM, cerebral malaria; MOD, multi-organ dysfunction; NCSM, non-cerebral severe malaria; UM, uncomplicated malaria; HC, healthy controls; S.D: standard deviation*.

### DNA isolation and genotyping of MBL polymorphisms

Genomic DNA was isolated from whole blood by Gen Elute Blood Genomic DNA Kit (Sigma Chemicals) according to manufacturer's instructions. MBL promoter polymorphisms (−550 H/Land −221 Y/X) and codon 54 polymorphism (A/B) were typed by double amplification refractory system (dARMS) and allele specific polymerase chain reaction (AS-PCR) respectively, as described earlier by us (Panda et al., 2013). MBL codon 52 and 57 were not included in the present investigation as these mutations are absent in studied population (Panda et al., 2013). PCR products were separated with 3% agarose gel electrophoresis, stained with ethidium bromide and visualized under UV light. About 30% of the randomly selected samples were sequenced. Results were found to be 100% concordant with genotyping by PCR, which ensured absence of genotyping error.

### MBL quantification

The plasma MBL was quantified by enzyme linked immnosorbentassay (ELISA) kit (R&D Systems) according to manufacturer's instructions.

### Statistical analysis

Genotype and allele frequency were calculated by direct counting. Web based Microsoft Excel tools was employed to calculate Hardy-Weingberg equilibrium (http://www.tufts.edu/~mcourt01/Documents/Court%20lab%20-%20HW%20calculator.xls). Fisher's test was used for comparison of genotype, allele frequencies in different clinical categories of *P. falciparum* malaria. Odds ratios (ORs), 95% confidence intervals (95% CIs) were calculated by Graphpad prism 5.01. P value less than 0.01 was taken assignificant (Bonferroni correction for three SNPs 0.05/3 = 0.01). Mean plasma MBL levels in various clinical categories were compared by analysis of variance (ANOVA) followed by Turkey's multiple comparison post-test.

## Results

### Estimation of plasma MBL levels in *P. falciparum* infected patients and healthy controls

**Figure 1:**
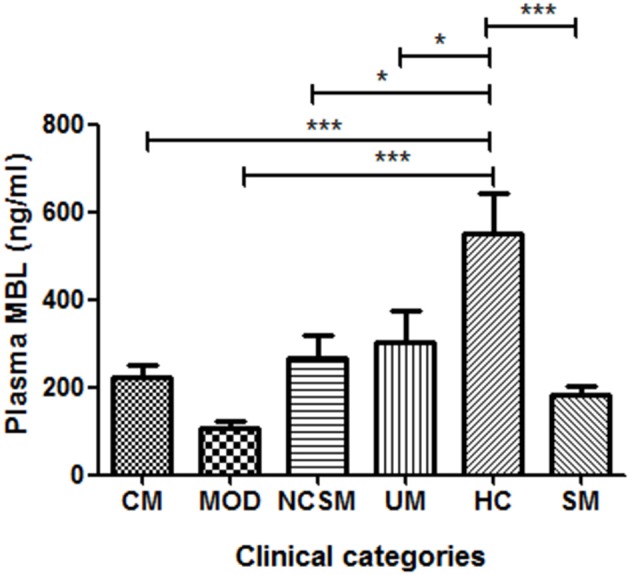
**Plasma MBL levels in different clinical categories of** ***P.falciparum*** **malaria and healthy controls**. Plasma samples from severe malaria (SM) patients [*n* = 47: CM (*n* = 17), MOD (*n* = 20), NCSM (*n* = 10)], UM (*n* = 12) and HC (*n* = 25) were quantified by ELISA according to manufacturer's instructions. Mean plasma levels of MBL among various clinical categories were compared by ANOVA followed by turkey's multiple comparison post-test. *P*-value less than 0.05 was considered as significant. ^*^P < 0.05; ^**^P < 0.01; ^***^P < 0.001.

As shown in Figure 1, healthy controls showed significantly higher levels of plasma MBL compared to severe malaria (*P* < 0.001) and uncomplicated malaria cases (*P* < 0.05). Furthermore, on analyses of the subtypes of SM, namely, CM, MOD, and NCSM, MOD patient showed least plasma MBL levels (107.1 ± 17.09 ng/ml) than CM (223.4 ± 27.66) and NCSM (268.5 ± 49.25). However, mean plasma MBL levels among different clinical categories of SM patients were not statistically significant. Mean plasma levels of MBL in HC was significantly higher compared to CM (*P* < 0.001), MOD (*P* < 0.001), and NCSM (*P* < 0.05).

### Distribution of MBL polymorphism in healthy subjects

Among 100 healthy individuals, 15 were heterozygous and five were homozygous for codon 54 polymorphism. The prevalence of HL and LL genotypes of MBL (H-550L) polymorphism was found to be 25 and 69%, respectively. The distribution of YX and XX genotypes of MBL promoter (Y-221X) was 29 and 16% respectively in healthy individuals. Frequency of MBL polymorphisms (codon 54, H-550L, and Y-221X) were comparable to our earlier report (Panda et al., 2013), and genotype distribution of codon 54 and MBL (Y-221X) deviated from Hardy-Weinberg equilibrium [MBL 54 (A/B): χ^2^ = 9.87, *P* = 0.001; MBL (H-550L): χ^2^ = 2.92, *P* = 0.08; MBL (Y-221X): χ^2^ = 9.98, *P* = 0.001].

### Genotype-phenotype association of MBL polymorphisms

**Figure 2:**
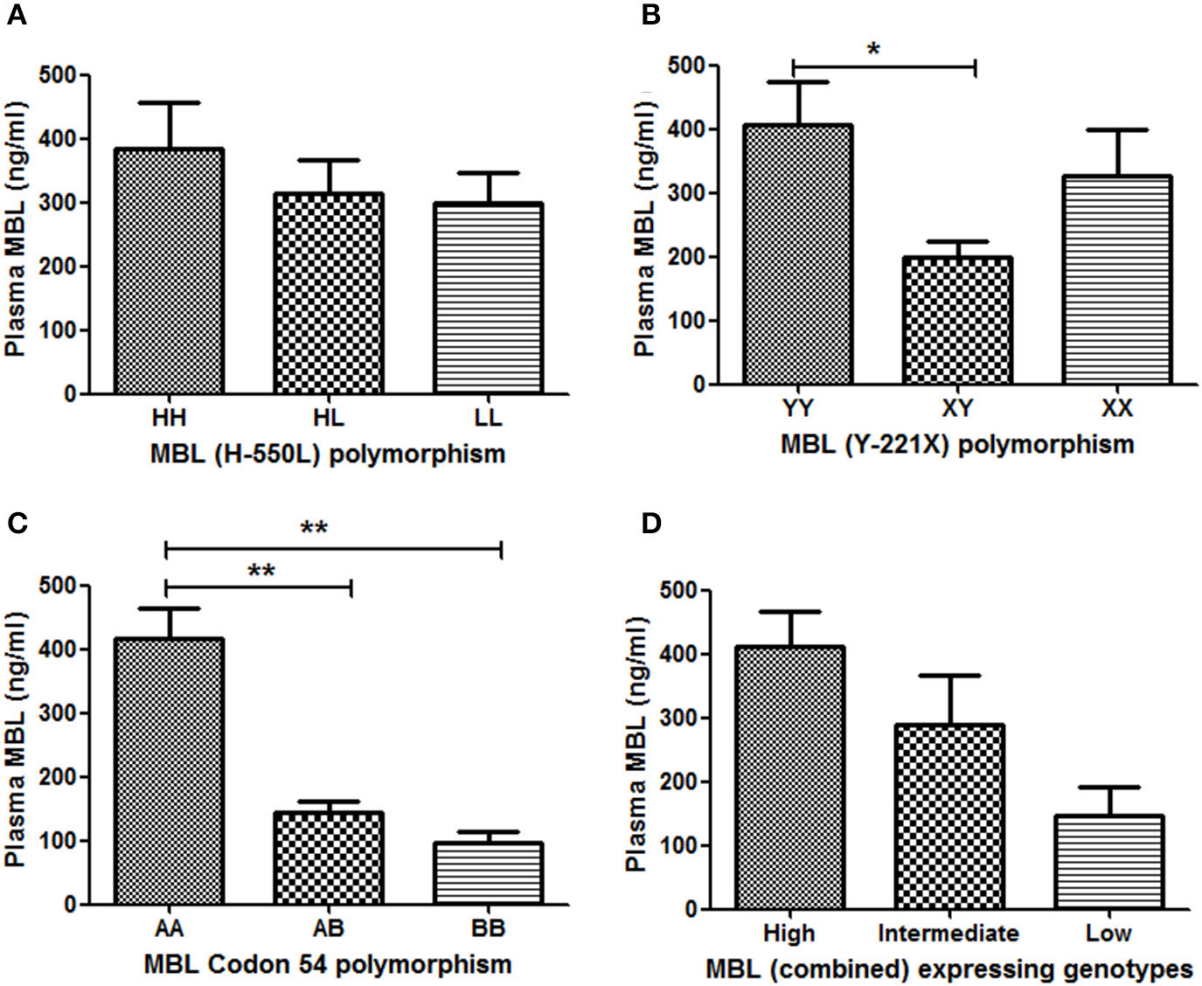
**Association between** ***MBL2*** **polymorphisms and plasma MBL levels**. Plasma concentrations (mean ± standard deviation) of MBL were measured, using a commercial kit. Based on availability, plasma of 84subjects were quantified and correlated with their respective genotypes (**A**: codon 54 A/B, **B**: Y-221X, and **C**: H-550L). Combined genotypes were grouped in to higher (HYA/HYA, HYA/LYA, HYA/LXA, LYA/LYA, and LYA/LXA), intermediate (HYA/LYB, LYA/LYB, and LXA/LXA) and lower expressing (LXA/LYB, LYB/LYB, and LXB/LXB) genotypes **(D)**. Mean plasma levels of MBL among genotypes were compared by ANOVA followed by turkey's multiple comparison post-test. *P*-value less than 0.05 was considered as significant. ^*^P < 0.05; ^**^P < 0.01.

Out of 567 subjects, plasma samples of 84 were available for MBL quantification by ELISA [CM (*n* = 17); MOD (*n* = 20); NCSM (*n* = 10); UM (*n* = 12); and HC (*n* = 25)]. Association between MBL2 polymorphisms and plasma levels of MBL are shown in Figure 2. YY genotype of MBL (Y-221X) polymorphism displayed significantly higher levels of plasma MBL compared to XY (Figure 2B). Furthermore, a significant association of MBL codon 54 polymorphism with plasma MBL was observed: wild type (AA) displayed higher MBL levels compared to AB (*P* < 0.01) and BB (*P* < 0.01) genotypes (Figure 2C). However, other promoter polymorphism (H-550L) failed to show an association with plasma levels of MBL (Figure 2A).

Plasma MBL level is believed to be controlled by the combined effect of promoter and codon polymorphisms. Earlier we had shown an association of MBL combined genotype with plasma levels of MBL (Panda et al., 2013). Combined genotypes were grouped as high producer (HYA/HYA, HYA/LYA, HYA/LXA, LYA/LYA, and LYA/LXA), intermediate producer (HYA/LYB, LYA/LYB, and LXA/LXA) and low producer (LXA/LYB, LYB/LYB, and LXB/LXB) of MBL, and the plasma levels of MBL were quantified in each group. High expressing genotypes displayed higher levels of plasma MBL than intermediate expressing genotypes. The low expressing genotypes was associated with lowest levels of plasma MBL. However, the differences of mean plasma MBL levels between these groups (high, intermediate and low) were not statistically significant.

### Codon 54 (A/B) and promoter (Y-221X) polymorphism are associated with MOD

Association of *MBL2* polymorphism with various clinical manifestation of *P. falciparum* malaria was analyzed by Fisher exact test. As depicted in Table 2, frequency of MBL codon 54 heterozygous (AB) and minor allele (B) were significantly higher in multi organ dysfunction compared to uncomplicated malaria patients (AB: *P* = 0.006, OR = 2.34, 95% CI = 1.26 to 4.33; B: *P* = 0.008, OR = 1.91, 95% CI = 1.19 to 3.09). Furthermore, dominant inheritance model comparison reveled a significant association with susceptibility of MOD (*P* = 0.005, OR = 2.23, 95% CI = 1.27 to 3.91). Heterozygous (YX) for *MBL* promoter polymorphism (Y-221X) was more frequent in MOD cases than UM (*P* = 0.009, OR = 2.06, 95% CI = 1.19 to 3.56). Distribution of MBL2 (H-550L) genotypes were comparable among different clinical categories of *P. falciparum* malaria. Prevalence of *MBL2* codon 54 heterozygous (AB), minor allele (B) and heterozygous for *MBL2* (Y-221X) polymorphism (YX) was higher in severe malaria compared to UM, but these differences could not reach statistically significance levels after Bonferroni correction.

**Table 2:** **Distribution of MBL (codon 54, H-550L, and Y-221X) polymorphisms in** ***P. falciparum*** **malaria**.

**MBL 2 polymorphism**		**Clinical categories, %V of subject**				**CM vs. UM**		**MOD vs. UM**		**NCSM vs. UM**		**SM vs. UM**	
	**CM (*n* = 94)**	**MOD (*n* = 120)**	**NCSM (*n* = 122)**	**SM (*n* = 336)**	**UM (*n* = 131)**	**OR (95%CI)**	***P*-value**	**OR (95%CI)**	***P*-value**	**OR (95%CI)**	***P*-value**	**OR (95%CI)**	***P*-value**
**MBL 54 (A/B)**													
Co-dominant inheritance													
AA	64 (68)	76 (63)	91 (75)	231 (69)	104(80)	1	Ref	1	Ref	1	Ref	1	Ref
AB	25 (27)	36 (30)	24 (20)	85 (25)	21 (16)	1.93 (1.00to3.73)	0.06	**2.34 (1.26 to 4.33)**	**0.006**	1.30 (0.68to2.50)	0.50	1.82 (1.07to3.09)	0.02
BB	5 (5)	8 (7)	7 (5)	20 (6)	6 (4)	1.35 (0.39to4.62)	0.75	1.77 (0.59to5.33)	0.40	1.33 (0.43to4.11)	0.77	1.50 (0.58to3.84	0.50
Dominant Inheritance													
AA	64 (68)	76 (63)	91 (75)	231 (69)	104(80)	1	Ref	1	Ref	1	Ref	1	Ref
AB+BB	30 (32)	44 (37)	31 (25)	105 (31)	27 (20)	1.80 (0.98to3.31)	0.06	**2.23 (1.27 to 3.91)**	**0.005**	1.31 (0.72to2.36)	0.37	1.75 (1.08to2.83)	0.02
Recessive inheritance													
AA+AB	89 (95)	112 (93)	115 (95)	316 (94)	125 (96)	1	Ref	1	Ref	1	Ref	1	Ref
BB	5 (5)	8 (7)	7 (5)	20 (6)	6 (4)	1.17 (0.34to3.95)	1.00	1.48 (0.50to4.42)	0.58	1.26 (0.41to3.88)	0.77	1.31 (0.51to3.36)	0.65
Allele													
A	153 (81)	188 (78)	206 (84)	547 (81)	229 (87)	1	Ref	1	Ref	1	Ref	1	Ref
B	35 (19)	52 (22)	38 (16)	125 (19)	33 (13)	1.58 (0.94to2.66)	**0.08**	**1.91 (1.19 to 3.09)**	**0.008**	1.28 (0.77to2.11)	0.37	1.58(1.04to2.39)	0.03
**H-550L**													
Co-dominant inheritance													
HH	5 (5)	7 (6)	9 (7)	21 (6)	7 (5)	1	Ref	1	Ref	1	Ref	1	Ref
HL	24 (26)	30 (25)	33 (27)	87 (26)	33 (25)	1.01 (0.28to3.59)	1.00	0.90 (0.28to2.89)	1.00	0.77 (0.25to2.33)	0.78	0.87 (0.34to2.26)	1.00
LL	65 (69)	83 (69)	80 (66)	228 (68)	91 (70)	1.00 (0.30to3.29)	1.00	0.91 (0.30to2.71)	1.00				
Dominant inheritance													
HH	5 (5)	7 (6)	9 (7)	21 (6)	7 (5)	1	Ref	1	Ref	1	Ref	1	Ref
HL+LL	89 (95)	113 (94)	113 (93)	315 (94)	124 (95)	1.00 (0.30to3.26)	1.00	0.91 (0.30to2.67)	1.00	0.70 (0.25to1.96)	0.60	0.84 (0.35to2.04)	0.83
Recessive inheritance													
HH+HL	29 (31)	37 (31)	42 (34)	108 (32)	40 (30)	1	Ref	1	Ref	1	Ref	1	Ref
LL	65 (69)	83 (69)	80 (66)	228 (68)	91 (70)	0.98 (0.55to1.75)	1.00	0.98 (0.57to1.68)	1.00	0.83 (0.49to1.41)	0.59	0.92 (0.59to1.43)	0.82
Allele													
H	34 (18)	44 (18)	51 (21)	129 (19)	47 (18)	1	Ref	1	Ref	1	Ref	1	Ref
L	154 (82)	196 (82)	193 (79)	543 (81)	215 (82)	0.99 (0.60to1.61)	1.00	0.97 (0.61to1.53)	0.90	0.82 (0.53to1.28)	0.42	0.92 (0.63to1.33)	0.70
**Y-221X**													
Co-dominant inheritance													
YY	43 (46)	50 (42)	63 (52)	156 (46)	72 (55)	1	Ref	1	Ref	1	Ref	1	Ref
YX	38 (40)	56 (47)	44 (36)	138 (41)	39 (30)	1.63 (0.90to2.92)	0.10	**2.06 (1.19 to 3.56)**	**0.009**	1.28 (0.74to2.23)	0.40	1.63 (1.03to2.56)	0.03
XX	13 (14)	14 (11)	15 (12)	42 (13)	20 (15)	1.08 (0.49to2.40)	0.84	1.00 (0.46to2.18)	1.00	0.85 (0.40to1.81)	0.70	0.96 (0.53to1.76)	1.00
Dominant inheritance													
YY	43 (46)	50 (42)	63 (52)	156 (46)	72 (55)	1	Ref	1	Ref	1	Ref	1	Ref
YX+XX	51 (54)	70 (58)	59 (48)	180 (54)	59 (45)	1.44 (0.85to2.46)	0.17	1.70 (1.03to2.81)	0.04	1.14 (0.69to1.87)	0.61	1.40 (0.93to2.11)	0.10
Recessive inheritance													
YY+YX	81	106	107	294	111 (85)	1	Ref	1	Ref	1	Ref	1	Ref
XX	13 (14)	14 (11)	15 (12)	42 (13)	20 (15)	0.89 (0.41to1.89)	0.84	0.73 (0.35to1.52)	0.46	0.77 (0.37to1.59)	0.58	0.79 (0.44to1.41)	0.44
Allele													
Y	124 (66)	156 (65)	170 (70)	450 (67)	183 (70)	1	Ref	1	Ref	1	Ref	1	Ref
X	64 (34)	84 (35)	74 (30)	222 (33)	79 (30)	1.19 (0.80to1.78)	0.41	1.24 (0.85to1.81)	0.25	1.00 (0.68to1.47)	1.00	1.14 (0.83to1.55)	0.43

*Data are no. (%) of participants unless otherwise specified. SM, severe malaria; CM, cerebral malaria; MOD, multi-organ dysfunction; NCSM, non-cerebral severe malaria; UM, uncomplicated malaria. Bold values are values which showed statistical significance*.

To find out association of *MBL2* polymorphism with mortality due to *P. falciparum* infection, distribution of codon 54, H-500L, Y-221X and combined genotypes was analyzed among subjects those died and survivors. No significant association of promoter, codon 54 polymorphism or combined genotypes was observed with malarial mortality (Data not shown).

### Low MBL producer genotypes are associated with MOD

As plasma MBL levels correlated with combined MBL2 genotypes, we analyzed distribution of MBL2 combined genotype in different clinical manifestations of *P. falciparum* malaria. As shown in Table 3, the prevalence of low MBL producer genotypes (LXA/LYB, LYB/LYB, LXB/LXB) were significantly higher in severe malaria patients (*P* = 0.009, OR = 3.04, 95% CI = 1.25 to 7.37) and multi organ dysfunction (*P* = 0.003, OR = 4.03, 95% CI = 1.56 to 10.52) compared to uncomplicated malaria.

**Table 3:** **Combined functional genotypes distribution of** ***MBL2*** **(promoter and exon1) polymorphisms in different clinical categories of** ***P. falciparum*** **malaria**.

**MBL2 combined genotype**	**Clinical categories, % of subject**					**CM vs. UM**		**MOD vs. UM**		**NCSM vs. UM**		**SM vs. UM**	
** **	**CM (*n* = 94)**	**MOD (*n* = 120)**	**NCSM (*n* = 122)**	**SM (*n* = 336)**	**UM (*n* = 131)**	**OR (95% CI)**	***P*-value**	**OR (95% CI)**	***P*-value**	**OR (95% CI)**	***P*-value**	**OR (95% CI)**	***P*-value**
High MBL producer	62 (66)	81 (67)	88 (72)	231 (69)	98 (75)	1	ref	1	Ref	1	ref	1	ref
Intermediate MBL producer	21 (22)	19 (16)	22 (18)	62 (18)	27 (21)	1.22 (0.63 to 2.36)	0.61	0.85 (0.44 to 1.64)	0.73	0.90 (0.48 to 1.70)	0.87	0.97 (0.58 to 1.62)	1.00
Low MBL producer	11 (12)	20 (17)	12 (10)	43 (13)	6 (5)	2.89 (1.02 to 8.23)	0.06	**4.03 (1.56 to 10.52)**	**0.003**	2.22 (0.80 to 6.18)	0.14	3.04 (1.25 to 7.37)	0.009

*Data are no. (%) of participants unless otherwise specified. High MBL producer: HYA/HYA, HYA/LYA, HYA/LXA, LYA/LYA, LYA/LXA; Intermediate MBL producer: HYA/LYB, LYA/LYB, LXA/LXA; Low MBL producer: LXA/LYB, LYB/LYB, LXB/LXB.OR, odds ratio, CI, confidence interval. Bold values are values which showed statistical significance*.

#### Correlation of plasma MBL levels with clinical parameters

We analyzed the possible correlation between plasma MBL levels and various clinical parameters and results are shown in Table 4. The plasma MBL levels had an inverse correlation with SGPT (*P* = 0.005, *r* = −0.46), serum urea (*P* = 0.03, *r* = −0.28) and alkaline phosphatase (*P* = 0.01, *r* = −0.34). These observations appear to be associational rather than a cause and effect relationship.

**Table 4:** **Correlation of Plasma MBL levels with clinical parameters of** ***P. falciparum*** **malaria**.

	***P*-value**	**Spearman *r***	**95% Confidence interval**
Hemoglobin	0.20	0.17	−0.09 to 0.41
SGOT	0.83	0.03	−0.31 to 0.38
SGPT	**0.005**	**−0.46**	−**0.70 to** −**0.14**
Serum Urea	**0.03**	**−0.28**	−**0.52 to** −**0.009**
Serum creatinine	0.05	−0.27	−0.51 to 0.01
Alkaline phosphatase	**0.01**	**−0.34**	−**0.56 to** −**0.07**

*Bold values are values which showed statistical significance*.

## Discussion

In the present case control study, we investigated the role of MBL in *P. falciparum* malaria in an endemic region of Odisha, India. Our data suggest that low levels of MBL and MBL2 low producer genotypes are associated with multi organ dysfunction. Furthermore, in the current investigation we also confirm a correlation between *MBL2* polymorphism with plasma levels of MBL.

MBL is an important component of the innate immune system. It plays a significant role in the defense against various infections. It recognizes carbohydrate domain on the microbial surface, opsonizes the pathogen and/or activates lectin pathways (Turner, 1996). Role of MBL in malaria has been demonstrated earlier (Luty et al., 1998; Juliger et al., 2002; Thevenon et al., 2009). In the present study, we observed a significantly higher level of plasma MBL in healthy controls from the endemic areas compared to uncomplicated and severe malaria patients, indicating a possible role for plasma MBL in the defense against *P. falciparum* infection. MBL binds to infected erythrocytes (Garred et al., 2003b) and possibly assists in the clearance of infected cells. Individuals with lower plasma MBL levels may have an increased susceptibility to malarial infection due to lack of this mechanism, and the infection may progress to develop severe disease. However, previous reports from the African continent indicate that normal healthy controls have lower MBL levels compared to patients with severe malaria (Luty et al., 1998) and *P. falciparum* infected cases (Juliger et al., 2002). Our study deals with adult patients with clinically distinct clinical phenotypes of severe malaria from an endemic area that has seasonal transmission. The earlier studies were from geographically different areas with distinct population base and transmission dynamics (Luty et al., 1998; Juliger et al., 2002). Children are mostly susceptible to severe malaria in Africa and they have a different clinical profile while adults do not suffer from severe disease. The discordance of observation in our study and the earlier reported data could be due to the difference in the population studied. Among the various categories of severe malaria patients, MOD patients had lower plasma MBL levels compared to CM and NCSM. MOD has high mortality among those with severe malaria. Serum MBL levels appear to modulate severity of disease by mechanisms which are not clearly defined.

Although large numbers of SNPs in MBL2 gene have been reported, only limited SNPs have functional relevance. Variants at amino acids coding region may lead to deformed proteins which degrade leading to its lower levels. Mutation at promoter regions may similarly affect binding of transcription factors leading to diminished production of transcripts which ultimately translate to lesser protein expression. Our study revealed an association of BB and AB genotypes with lower plasma MBL similar to studies reported earlier in Danish (Madsen et al., 1995; Steffensen et al., 2000), Australian (Minchinton et al., 2002), and Indian population (Panda et al., 2013). We also observed association between *MBL2* Y-221X polymorphism with plasma MBL levels similar to our earlier report (Panda et al., 2013). However, MBL2 combined genotype and H-550L polymorphism did not correlate with plasma levels of MBL. This could be a result of small sample size available for plasma MBL estimation.

We observed a significant association between *MBL2* codon 54 and promoter Y-221X polymorphism with susceptibility to multi organ dysfunction. Subjects with heterozygote genotype for codon 54 (AB) and Y-221X (YX) polymorphism had 2.34 and 2.06 fold higher chance of developing MOD. Furthermore, combined analysis of codon and promoter polymorphism revealed significant association of low producer genotype (LXA/LYB, LYB/LYB, LXB/LXB) with susceptibility to MODs (OR = 4.03). Earlier studies reported the role of MBL2 polymorphism in severe malaria. *MBL2* codon 54 and 57 variants in Gabonese children, LXPA haplotype in Indian population (Jha et al., 2014) and a novel mutation (−797 C>A) (Boldt et al., 2006) have been linked with susceptibility to severe malaria. The present study also showed similar susceptibility of *MBL2* codon 54 and Y-221X variants to severe malaria but did not reach significance after Bonferoni correction.

In the present investigation, distribution of MBL codon 54 (A/B) and promoter (H-550L) polymorphism deviated from HWE in line with our earlier observation (Panda et al., 2013). Disparity of genotype distribution from HWE has been attributed to population stratifications and/or selection pressure (Hosking et al., 2004). In the current study, patients and controls were enrolled from a similar geographical area, and the possible reason for deviation of HWE could be selection pressure. Malaria exerts a strong selection pressure on the human genome and beneficial allele are positively selected and become more prevalent in endemic areas. The studied population is also endemic to filariasis, and a previous study by our group (unpublished results), and others (Choi et al., 2001; Meyrowitsch et al., 2010) had shown an association between *MBL2* variants with susceptibility to lymphatic filariasis. Infectious diseases exert selection pressure on human population (Miller, 1999; Ghosh, 2008; Fumagalli et al., 2009) and parasite endemic areas are significantly affected.

Our study clearly defines the association between low plasma levels of MBL and the low producer MBL genotype with severe disease, notably, MOD. Although it is known that MBL functions as an important protective component of the innate immune system, the mechanism(s) that modulates severity in *P. falciparum* remains to be clearly defined.

## Authors' contributions

AP was involved in design, performing experiments, analysis, interpretation, performed statistics, and writing the first draft of the manuscript. BD made a contribution in the design, data interpretation, work supervision and critically revising the manuscript. All authors read and approved the manuscript.

### Conflict of interest statement

The authors declare that the research was conducted in the absence of any commercial or financial relationships that could be construed as a potential conflict of interest.
